# Extended Release Combination Antibiotic Therapy from a Bone Void Filling Putty for Treatment of Osteomyelitis

**DOI:** 10.3390/pharmaceutics11110592

**Published:** 2019-11-08

**Authors:** Raquib Hasan, Kambri Schaner, Meredith Schroeder, Abbey Wohlers, Jacob Shreffler, Codi Schaper, Hariharaputhiran Subramanian, Amanda Brooks

**Affiliations:** 1 Department of Pharmaceutical Sciences, North Dakota State University, Fargo, ND 58102, USA; raquib.hasan@ndsu.edu (R.H.); jacob.shreffler@ndsu.edu (J.S.); 2 Department of Biological Sciences, North Dakota State University, Fargo, ND 58102, USA; kambri.schaner@ndsu.edu; 3 Department of Microbiological Sciences, North Dakota State University, Fargo, ND 58102, USA; meredith.schroeder@ndus.edu; 4 Department of Pharmacy, North Dakota State University, Fargo, ND 58102, USA; abbey.wohlers@ndsu.edu; 5 Department of Animal Sciences, North Dakota State University, Fargo, ND 58102, USA; codischaper@gmail.com; 6 Department of Chemistry and Biochemistry, North Dakota State University, Fargo, ND 58102, USA; hariharaputhiran.sub@ndsu.edu

**Keywords:** osteomyelitis, total joint replacement, biofilm, infection, vancomycin, rifampicin, combination antibiotic delivery, bone void filler, drug release

## Abstract

In spite of advances in Total Joint Replacements (TJR), infection remains a major concern and a primary causative factor for revision surgery. Current clinical standards treat these osteomyelitis infections with antibiotic-laden poly(methyl methacrylate) (PMMA)-based cement, which has several disadvantages, including inadequate local drug release kinetics, antibiotic leaching for a prolonged period and additional surgical interventions to remove it, etc. Moreover, not all antibiotics (e.g., rifampicin, a potent antibiofilm antibiotic) are compatible with PMMA. For this reason, treatment of TJR-associated infections and related complications remains a significant concern. The objective of this study was to develop a polymer-controlled dual antibiotic-releasing bone void filler (ABVF) with an underlying osseointegrating substrate to treat TJR implant-associated biofilm infections. An ABVF putty was designed to provide sustained vancomycin and rifampicin antibiotic release for 6 weeks while concurrently providing an osseointegrating support for regrowth of lost bone. The reported ABVF showed efficient antibacterial and antibiofilm activity both in vitro and in a rat infection model where the ABVF both showed complete bacterial elimination and supported bone growth. Furthermore, in an in vivo k-wire-based biofilm infection model, the ABVF putty was also able to eliminate the biofilm infection while supporting osseointegration. The retrieved k-wire implants were also free from biofilm and bacterial burden. The ABVF putty delivering combination antibiotics demonstrated that it can be a viable treatment option for implant-related osteomyelitis and may lead to retention of the hardware while enabling single-stage surgery.

## 1. Introduction

Although Total Joint Replacements (TJR), including Total Knee Replacements (TKR) and Total Hip Replacements (THR), are arguably one of the most successful surgical interventions to improve quality of life, there is a growing trend in the number of secondary or revision surgeries being done in the United States, according to the American Academy of Orthopedic Surgeons (AAOS). More than a million people undergo Total Joint Replacements (TJR) each year in the United States currently; with projections of more than 4 million procedures a year by 2030 [1,2,3], of which, an estimated 365,000 will be secondary or revision procedures [2]. These numbers do not necessarily reflect a failure in the TJR procedure as much they are an indication of (1) surgical intervention at an earlier age [4,5] often necessitating a revision surgery later in life as the TJR implants materials wear out and (2) rising obesity leading to osteoarthritis; osteoarthritis is reported to account for >90% of Total Knee Replacements (TKR) procedures, and (3) infection. Despite its low incidence in primary TJR (1–2%), infection is perhaps the most concerning cause for TJR revision, accounting for 18% and 26.8% of revision THR and TKR, respectively. Responsible for ~65% of total cases, *Staphylococcus aureus* is the most common causative pathogen-associated prosthetic joint infection (PJI) after TJR. Almost 20% of these *S. aureus* infections are classified as methicillin resistant (MRSA) [6,7]. Not only do these infected TJR surgeries impose a significant socioeconomic and mortality burden, they also predispose the implant and surrounding bone to further threats from infection, driving the risk of recurrence to a staggering 21%. Implant removal and hardware replacement can propel the risk of infection to a threatening 20–30% [8,9]. Importantly, non-infected revision TJR also has a modestly increased infection risk of 5.4% [10].

Osteomyelitis, or deep bone infection, is clinically difficult to treat, often due to the development of a biofilm associated with the implanted hardware [11] and infiltration of the surrounding soft tissue with bacteria [12]. The Centers for Disease Control estimates that 80% of all bacterial infections are caused by biofilms, including those associated with medical devices such as joint prostheses, catheters, and heart valves [13]. Biofilm resident bacteria often require up to 1000-fold more antibiotic to eradicate the infection when compared to their planktonic counterparts [14]. The current clinical treatment regimen for revision TJR, particularly those due to infection, centers around implantation with antibiotic-loaded Polymethylmethacrylate (PMMA) bone cement for local antibiotic delivery after thorough debridement of infected and necrotic tissue. Importantly, due the leaching of low concentration antibiotic from PMMA bone cements over an extended period of time, antibiotic-releasing PMMA can be associated with a higher incidence of localized antibiotic resistant bacteria [15].

Alternatively, a concurrent or sequential extended course of systemic antibiotics may also be used [16]. Often, vancomycin is the physician’s chosen systemic treatment for osteomyelitis due to the threat of methicillin-resistant *Staphylococcus aureus* (MRSA) [17], despite the risk of nephrotoxicity and red-man syndrome [18] associated with high-dose intravenous therapy. Unfortunately, not only does systemic vancomycin have poor bone penetration, with only a fraction of the drug reaching the bone to provide a bone/serum ratio of 10% and an even lower penetration in osteomyelitic bone [19], vancomycin also has limited activity against bacterial biofilms [20]. Alternatively, rifampicin, which has a variable ability to penetrate bone (from 8% to 56%), has demonstrated superior activity against both biofilm and slow growing bacteria [21]. In multiple clinical trials, the use of rifampicin in conjunction with another antibiotic resulted in infection cure and implant salvage in the majority of cases [12,22]. Importantly, monotherapy with rifampicin, particularly as a prolonged oral therapy (up to 6 months), was ill-advised as there was a higher possibility of resistant infection [21] and toxicity (i.e., gastrointestinal, hepatic, renal symptoms and red-man syndrome [23]) in patients. Despite its potential for biofilm infections, the local delivery of rifampicin, specifically from PMMA, has been hindered by the fact that rifampicin can act as a scavenger of reactive oxygen species, thereby preventing the polymerization of PMMA [24]. The utility of rifampicin as an adjunct antibiotic therapy combined with the uselessness of PMMA bone cement to locally deliver rifampicin to bone necessitates the development of an alternative antibiotic-releasing bone void filler (ABVF) with the potential to treat biofilm infections. Moreover, developing a biodegradable bone void filler with degradation matched antibiotic release kinetics can provide scaffold like support to guide bone tissue regeneration [25]. 

The current study evaluated not only the in vitro kinetics of dual vancomycin and rifampicin release from a developed press-fitting ABVF putty but also the in vitro antibacterial and anti-biofilm activity of the released combination. Additionally, in vivo host bone integration and antibiotic protection of the ABVF putty was evaluated in a pilot rat model of biofilm-seeded osteomyelitis. Both in vitro and in vivo results support the use of our developed ABVF putty for the local delivery of vancomycin and rifampicin to combat biofilm-associated osteomyelitis while avoiding systemic side effects. This effective osteomyelitis treatment will increase the rate of implant salvage, thereby reducing the socioeconomic burden of TJR infections on our healthcare system.

## 2. Materials and Methods

### 2.1. Materials

Poly(d,l-lactide-*co*-glycolide) 90:10 (PLGA) (Polysciences, Inc., Warington, PA, USA); Polyethylene Glycol (5kD) (PEG) (Fluka, St. Louis, MO, USA) and Polycaprolactone (10 kD) (PCL-Sigma-Aldrich, St. Louis, MO, USA) were used as received. Rifampicin (EMD Millipore, Darmstadt, Germany), *N*-Methyl-2-pyrrolidone (NMP) (Fisher Sci, Pittsburg, PA, USA), Calcium Chloride (CaCl_2_) (EMD Chemicals Inc., Gibbstown, NJ, USA), alamarBlue (Bio Rad, Hercules, CA, USA), Kirschner wire (K-wire) (Key Surgical, Eden Prairie, MN, USA) were all used as received from the manufacturer. Vancomycin Hydrochloride (V-HCl) (Sagent Pharmaceuticals, Schaumburg, IL, USA) and PRO OSTEON 500R (kindly provided by Zimmer Biomet, Warsaw, IN, USA) were processed, as outlined below, after receipt.

### 2.2. Preparation of Vancomycin Free-Base (V-fb)

V-fb was prepared from V-HCl following previously published method [23]. Briefly, V-HCl was dissolved in water at a concentration of 70 mg/mL. To precipitate V-fb at pH 8.00, NaOH (3 N) was added until pH 8.00 was achieved. After incubation for 30 min at ambient temperature and humidity, the precipitated V-fb was centrifuged at 3000 rpm for 10 min, followed by sequential washes in 70% ethanol and methanol. Subsequently, V-fb was suspended in water, frozen and lyophilized.

### 2.3. Fabrication and In Vitro Characterization

#### 2.3.1. Preparation of ABVF

ABVF putty was prepared according to our previously published method with few modifications [26]. Briefly, Pro Osteon 500R was morselized using a mortar and pestle and sieved to yield a particle size distribution of 175 to 425 μm. An amount of 350 mg of Pro Osteon particles was soaked with agitation (100 rpm) in a 1 mL solution of V-HCl/rifampicin (80 mg/mL of vancomycin and 20 mg/mL rifampicin, in 50:50 water:DMSO solvent) for 1 h at room temperature. The solution was subsequently evaporated to dryness at 37 °C under vacuum. To fabricate the ABVF putty, PEG (21.2 mg) and PCL (42.5 mg), were melted and mixed at 65 °C. The prepared Pro Osteon particles were added to the molten polymers to create a homogenous mixture. Powdered V-fb (30 mg) and rifampicin (25.5 mg) were added to the mixture. PLGA (85.5 mg) dissolved in 200 μL of NMP and CaCl_2_ (22 mg) were combined with the molten mixture to create a homogenous putty base. Phosphate buffered saline (PBS) was added dropwise (20 μL) to the putty base to give a putty-like consistency. Subsequently, the putty mix was manually compressed into a 3D-printed mold to get the desired size and cylindrical shape (4 mm diameter × 3.5 mm height) for surgical implantation into a rat drill hole model.

#### 2.3.2. In Vitro Drug Release Kinetics

An ABVF putty cylinder was submerged in 2 mL of PBS (1X) release media and was incubated at 37 °C (*n* = 4). At different time points (day 1, day 3, day 7 and every week after that until week 6), release media was collected and replaced with fresh PBS. The amount of drug in the sample was assessed via HPLC analyses on a Waters e2695 separation module (254 nm) with an Empower workstation. The standard samples (Vancomycin, Vancomycin free-base, or Rifampicin dissolved in PBS) were separated on a XTERRA RP 18 5 μm, 4.6 × 250 mm column using 0.1% TFA in water and 0.1% TFA in acetonitrile as mobile phases.

#### 2.3.3. In Vitro Antibacterial Activity

The in vitro antibacterial activity of the released drugs was assessed against *Staphylococcus aureus* (ATCC 49230) using a Kirby-Bauer zone of inhibition (ZOI) assay [24]. Briefly, 100 μL of the collected drug release media was dried on filter paper disks (6.5 mm diameter) in a 96-well plate. An overnight culture of *S. aureus* was diluted in PBS to get a bacterial concentration of 10^7^ CFU/mL. The bacteria culture was streaked on LB (Luria-Bertani) agar plates and the filter paper disks with adsorbed drug were placed on the plates immediately. Agar plates were incubated for 20 h at 37 °C. The zone around the disk with no bacterial growth was measured using a digital caliper.

#### 2.3.4. In Vitro Antibiofilm Assay

Overnight culture of *S. aureus* was adjusted to OD_600_ of 0.5 in LB broth. An amount of 1 mL of adjusted bacterial culture was added to each well of a 24-well plate and was incubated for 24 h at 37 °C with gentle shaking (100 rpm) to create a mature biofilm. After incubation, the broth was aspirated, and the remaining bacterial biofilm was washed gently with 1X PBS three times. After washing, 500 µL of fresh broth and 500 µL of drug release media from day 1 was added to the wells. Control wells received only PBS and broth without drug. Wells were incubated for 24 h at 37 °C with gentle shaking (100 rpm), and the liquid was aspirated. Each well was again washed with 1X PBS three times. The bacteria in the remaining biofilm were extracted from the surface by pipetting with 1X PBS, followed by swabbing with cotton swabs. The swab was put into the collected PBS and vortexed for 1 min. The bacterial suspension was used for colony counts. Briefly, serial dilutions were made, and the bacteria were plated on LB agar plates. After incubating 20 h at 37 °C, bacteria colonies were counted. Alternatively, a similar procedure was followed with modifications to assess the antibiofilm capability of the drug released from the ABVF putty on biofilm grown on a k-wire. 

K-wire (1.4 mm diameter) was cut into 12 mm lengths and autoclaved. Subsequently, sterilized k-wires were immersed into LB broth and dried at 50 °C to produce a film-like coating of nutrients on the wire. An overnight culture of *S. aureus* was adjusted to OD_600_ = 0.5 in LB broth. To form a mature biofilm on the k-wire, the prepared k-wires were put into 24-well plates at an angle and 1.5 mL of bacterial culture was added to the wells to completely submerge the k-wires. After incubating for 24 h at 37 °C and 100 rpm, the media was removed and the k-wires were washed gently in 1X PBS three times, leaving bacteria attached to the adherent biofilm. After washing, 500 µL of fresh LB broth and 500 µL of drug release media from day 1 was added to the wells and the biofilm covered k-wires were incubated at 37 °C (100 rpm) for 24 h. In control wells, 500 µL of fresh broth and 500 µL 1X PBS without drug was added. After incubation, k-wires were removed and again washed gently in 1X PBS to remove loosely adherent bacteria. The wire was vortexed in 1 mL 1X PBS for 60 s to dislodge the biofilm and create a bacterial suspension. Serial dilutions of the suspension were made, and bacterial dilutions were plated on LB agar plates. After 20 h of incubation at 37 °C, bacterial colonies were counted.

#### 2.3.5. In Vitro Cytocompatibility

The cytocompatibility of the ABVF putty was assessed via a standard alamarBlue assay, according to the manufacturer’s protocol. Briefly, 10,000 MG-63 osteoblast cells (ATCC, Manassas, VA, USA) were seeded in each well of a 96-well plate. Cells were grown in Dulbecco’s Modified Eagle Media (DMEM) containing 10% fetal bovine serum (FBS) and 1% penicillin-streptomycin-fungizone (Lonza, Walkersville, MD, USA). Cells were incubated at 37 °C and 5% CO_2_. After reaching 60% confluence, 100 μL of release media from ABVF was added to each well. Control wells received 1X PBS only. After 48 h of incubation, cells were washed with 1X PBS three times. Fresh media was added to the wells followed by addition of alamarBlue to a 10% final concentration. Subsequently, wells were incubated at 37 °C for 4 h. After the incubation, absorbance was read at 570 nm and 600 nm (Spectramax m5, Molecular Devices, Downingtown, PA, USA). Cell viability was calculated using the following equation:

% cell viability = [(O2 × A1) − (O1 × A2)]/[(O2 × P1) − (O1 × P2)],(1)

where O1 = molar extinction coefficient (E) of oxidized alamarBlue (Blue) at 570 nm, O2 = E of oxidized alamarBlue at 600 nm, A1 = absorbance of test wells at 570 nm, A2 = absorbance of test wells at 600 nm, P1 = absorbance of positive growth control well (cells plus alamarBlue but no test agent) at 570 nm, P2 = absorbance of positive growth control well (cells plus alamarBlue but no test agent at 600 nm).

### 2.4. In Vivo Assessment

#### 2.4.1. Rat Osteomyelitis Model

Twenty-three skeletally mature male Sprague-Dawley rats (>350 g) divided into 4 cohorts (Table 1) were used for the in vivo studies. All the animal studies were conducted in a humane and ethical manner under protocols A16016 (approval date: 19 October 2015) and A19019 (approval date: 8 October 2018) approved by the North Dakota State University’s Institutional Care and Use Committee. Briefly, rats were anesthetized with isoflurane inhalation. Buprenorphine hydrochloride (0.01 mg/kg) was injected subcutaneously as an analgesic. The right hind leg was shaved and sterilized using repeated swabs of alcohol and iodine. A small incision of ~12 mm was made below the knee and over the tibial metaphysis. A 4.2 mm hole was drilled in the exposed bone until it penetrated the marrow space of the tibial metaphysis. *S. aureus* suspension of 10 μL containing 10^8^ CFU of bacteria in sterile saline was inoculated through the drill hole defect into the marrow space using a 25 μL Hamilton syringe, followed by implantation of the cylindrical ABVF putty into the drill hole. Alternatively, as a preliminary study to assess the in vivo antibiofilm activity, a biofilm-seeded k-wire (12 mm length × 1.4 mm diameter) prepared according to method 2.3.6 was pushed inside the marrow space after making a 4.2 mm diameter drill-hole in tibial metaphysis, followed by implantation of the ABVF putty. The incision was closed using a series of mattress sutures followed by application of surgical glue (Vetbond Tissue Adhesive, 3M, Maplewood, MN, USA). The control group underwent the same surgical procedure but received the putty without antibiotic, regardless of the form of bacterial inoculation (i.e., plantktonic or biofilm). Rats were monitored daily for signs of discomfort and infection. After 10 weeks, the rats were sacrificed by isoflurane overdose, and the tibia was harvested for further study. Importantly, in the preliminary study, cohorts implanted with a biofilm-seeded k-wire were kept for 6 weeks prior to sacrifice, bone was harvested and the k-wire was extracted from the bone for further analysis. 

#### 2.4.2. X-ray and Microcomputed Tomography (μ-CT)

Radiographic analyses of bone were done after disarticulating the limb and harvesting the bone following euthanasia. X-ray imaging was performed on an IDEXX CR Digital Radiography System (Westbrook, ME, USA) following standard protocols. Briefly, lateral and cranial-caudal radiographic images were obtained of each limb at mAs: 4 and kVp: 40. For μ-CT, the bone was hot-glued to a glass rod and placed into a GE Phoenix v|tome| xs X-ray computed tomography system with a 180 kV high power nanofocus X-ray tube xs|180 nf, high contrast GE DXR250RT flat panel detector, and molybdenum target (GE Sensing & Inspection Technologies GmbH, Wunstorf, Germany). One thousand projections were acquired at a voltage of 80 kV and a current of 300 µA. Voxel size was 6.4 µm. Acquired images were reconstructed into a volume data set using GE datos|x 3D computer tomography software Version 2.2 (GE Sensing & Inspection Technologies GmbH, Wunstorf, Germany). The reconstructed volume was then viewed and manipulated using VGStudio Max (Volume Graphics Inc., Charlotte, NC, USA).

#### 2.4.3. Bone Volume

Bone volume was determined based on the μ-CT scans using VGStudio Max (Volume Graphics Inc., Charlotte, NC, USA) software. A region of interest (ROI) was set on each of the scanned bones according to where the drill hole was made. The amount of regenerated bone was determined by adjusting the contrast to highlight the bone and comparing the ROI from the infection control group (cohort 1) and the treatment group (cohort 2). Regenerated bone was the bone in the ROI where the drill hole was made and was filled up by new bone.

#### 2.4.4. Histology

After euthanasia, the bone was harvested and fixed in 10% neutral buffered formalin for 72 h. Subsequently, bone was decalcified by immersing in an EDTA solution (10% solution at pH 7.4) for 2 weeks, with solution exchange every other day. Once the bone was decalcified, it was embedded in paraffin wax and sectioned (5 μm). Sections were mounted on glass slides and stained with hemotoxin and eosin stains (H&E stain—Scy Tek Lab., Logan, UT, USA) according to standard protocols. Briefly, the sections were deparaffinized in Clear Rite 3 (Thermo Fisher Scientific, Kalamazoo, MI, USA). Subsequently, tissue was rehydrated with a decreasing gradient of ethanol. After H&E staining, the tissue section was covered with a glass coverslip using synthetic resin. Stained slides were imaged at 40× using a MoticEasyScan Digital Slide Scanning microscope (Motic Digital Pathology, San Francisco, CA, USA).

#### 2.4.5. Bacterial Colony Count

Harvested bone was flash frozen in liquid nitrogen, followed by pulverization using a custom-made bone crusher. The pulverized bone was weighed and suspended in 500 μL of PBS. Serial dilutions were made and 10 μL of suspension was plated on CNA blood agar plates (Fisher Sci, Pittsburg, PA, USA). The plates were incubated for 48 h at 37 °C and bacterial colonies were counted. To determine the total number of bacteria in the bone, the number of colonies were multiplied by the dilution factor and normalized for per gram bone samples. For the extracted k-wires, the k-wire was put in 1 mL of PBS and vortexed for 1 min. Serial dilutions were made and 10 μL of suspension was plated on CNA blood agar plates (Fisher Sci, Pittsburg, PA, USA). The plates were incubated for 48 h at 37 °C and bacterial colonies were counted.

### 2.5. Statistical Analysis

The statistical package in Microsoft Excel 2016 was used for all calculations and statistical analyses. A student’s *t*-test using an α = 0.05 was done to determine statistical significance.

## 3. Results

### 3.1. In Vitro Drug Release Kinetics

**Figure 1:**
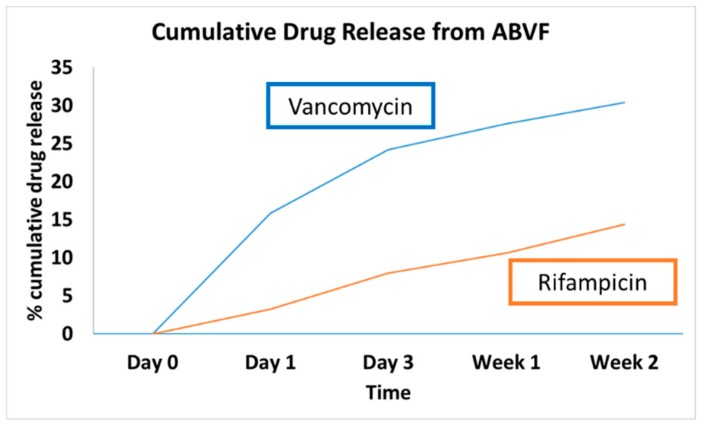
HPLC showed sustained release of vancomycin and rifampicin from ABVF. There was an initial burst release of vancomycin at day 1. Rifampicin showed release in a sustained manner.

HPLC detected both vancomycin and rifampicin in the release media through 2 weeks of release. ABVF showed sustained release of both drugs (Figure 1). Approximately 30% of the incorporated vancomycin was released within 2 weeks, while 14% of the rifampicin was released in the same timeframe. The current HPLC method was unable to detect drugs beyond week 2, likely due to interference from the degrading polymer matrix; hence, consideration of the bioactivity of the system may prove more useful.

### 3.2. In Vitro Antibacterial Activity

**Figure 2:**
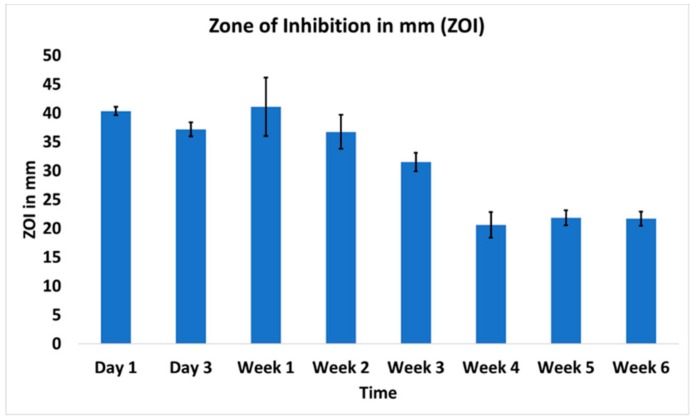
The zone of inhibition from the released drugs showed excellent antibacterial property against *S. aureus* (ATCC 49230). *n* = 4 for each time points.

Using a standard ZOI assay, efficient antibacterial activity was evident throughout the 6-week study time-course (Figure 2). Day 1 release samples showed the highest ZOI (40.35 ± 0.75 mm) against *S. aureus*. At week 6, the ZOI remained robust at 21.71 ± 1.23 mm. To ensure that sink conditions were maintained and did not impact the results, samples were also released in 5 mL of PBS and the ZOI study was replicated, yielding the same results (Supplementary Materials Figure S1). The concentrations of the released drugs were above the minimum concentration needed to show ZOI as compared to ZOIs of standards of vancomycin and rifampicin (Supplementary Materials Figure S2).

### 3.3. In Vitro Antibiofilm Assay

**Figure 3:**
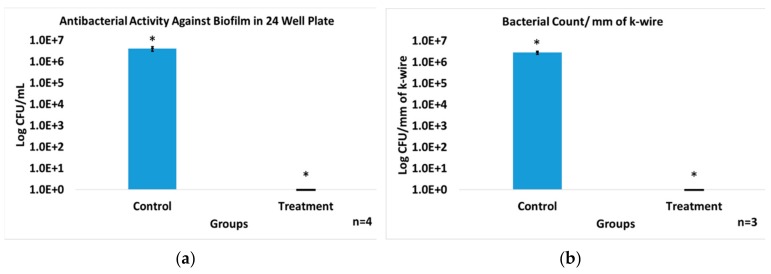
(**a**) The released drug showed excellent antibiofilm activity property against *S. aureus* (ATCC 49230) biofilm grown in a 24-well plate. The Treatment group (treated with day 1 release media) showed complete wipeout of the biofilm, whereas the control group that did not receive the release drug showed high bacterial content in the biofilm; *n* = 4, * *p* < 0.005. (**b**) *S. aureus* (ATCC 49230) biofilm grown on k-wire in vitro was completely eradicated in the treatment group treated with released antibiotics (day 1 release media) from the ABVF, while the control group showed a high bacterial count in the biofilm grown on k-wire; *n* = 3, * *p* < 0.002.

Since the majority of bacterial contaminants, particularly in the presence of biomedical implants, are found in a biofilm, the ability of the dual release ABVF was evaluated against a biofilm grown in well-plate. The biofilm was determined to have 4.05 × 10^6^ ± 9.81 × 10^5^ bacterial colony-forming units per mL (CFU/mL). Complete obliteration of the bacteria in the biofilm was seen when the biofilm was exposed to drug release milieu from ABVF, with no bacterial growth evident in a colony-forming assay. Alternatively, the control without antibiotics showed robust colony growth (*p* < 0.005, Figure 3a). Furthermore, the ability of a dual releasing ABVF to eliminate biofilm formation on a k-wire was also assessed. Biofilm formation on the k-wire was evident in the control group with 2.80 × 10^6^ ± 4.65 × 10^5^ CFU of bacteria per mm length of k-wire. Conversely, the treatment group samples showed no evidence of colony forming units in culture, indicating the effective eradication of viable bacteria in biofilm (Figure 3b).

### 3.4. In Vitro Cytocompatibility

**Figure 4:**
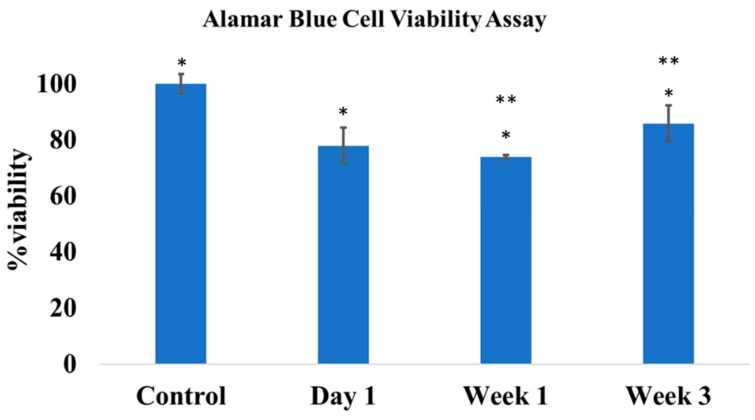
The alamarBlue cell viability assay showed a significant difference in cell viability between the control and the treatment groups with day 1, week 1 and week 3 drug release milieu (*p* < 0.05). There was also a significant difference between viability when cells were exposed to released media from week 1 and week 3 (*p* < 0.05), but no significant difference was seen between day 1 and week 1 and day 1 and week 3; *n* = 3 for each group, * *p* < 0.05, ** *p* < 0.05.

A standard alamarBlue assay was run to assess the cytotoxicity of the dual releasing ABVF putty on MG-63 osteoblasts. The viability of the treatment cells appeared to be less than the control cells, and a statistically significant difference (*p* < 0.05) was seen in cell viability (Figure 4). Cells exposed to week 3 release media showed significantly higher viability than week 1. This may indicate a lower amount of drug caused less impact on viability of the cells.

### 3.5. Rat Osteomyelitis Models

Infection was seen in all the animals of cohort 1 (i.e., the control cohort—Table 1). The macroscopic evaluation of the affected bone revealed purulent drainage and formation of sequestrum, including fibrous encapsulated, pus-filled regions in the tibial bone. The rats did not show any overt behavioral changes and did not lose weight over the experimental period (in fact they gained weight over the experimental period) and did not show any increase in body temperature. Importantly, spontaneous fracture was seen in three rats of cohort 1 within 3 days of surgery, and they were excluded from the study. None of the ABVF treatment rats in cohort 2 showed any signs of infection nor did they show any changes in behavior. Furthermore, they looked healthy and did not show any physiological signs of discomfort or limited mobility. In addition to assessing the dual releasing ABVF putty against planktonic bacteria, a preliminary assessment of the effect of the ABVF putty on a biofilm infection was also evaluated. As in the planktonic control cohort, infections developed in all cohort 3 control rats, as evidenced by formation of purulent pus, a pus-filled fibrous capsule, and deformed bone with thickening at different places. Again, no behavioral or physiological changes (i.e., weight loss or temperature increase) were observed. Healing without any signs of infection was observed in the cohort 4 animals containing ABVF with antibiotics. Similar to cohort 2, no behavioral changes were seen in the cohort 4 rats, and they gained weight over the experimental period while maintaining a normal body temperature of ~36 °C.

### 3.6. X-ray and Microcomputed Tomography (μ-CT)

**Figure 5:**
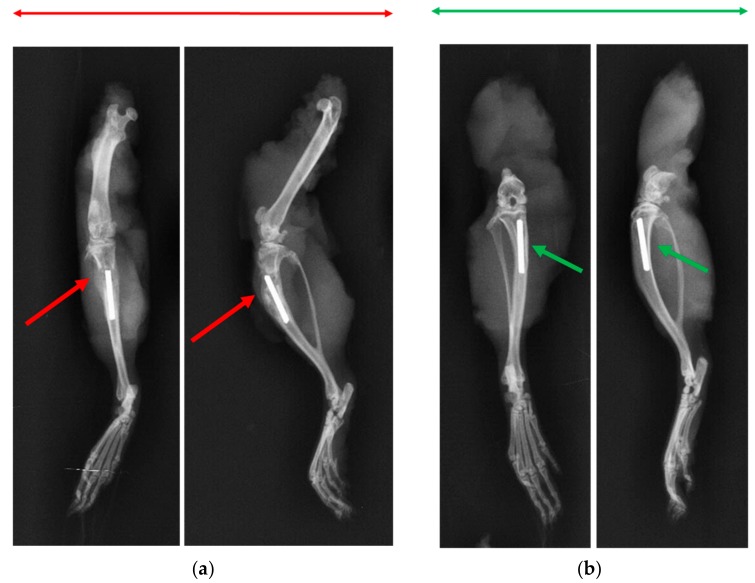
(**a**) The x-ray radiograph of the k-wire rat control group demonstrated signs of osteomyelitis (i.e., osteolysis, periosteal thickening and formation of sequestrum). (**b**) The k-wire treatment group showed no such signs of osteomyelitis and the bone appeared to be healed.

**Figure 6:**
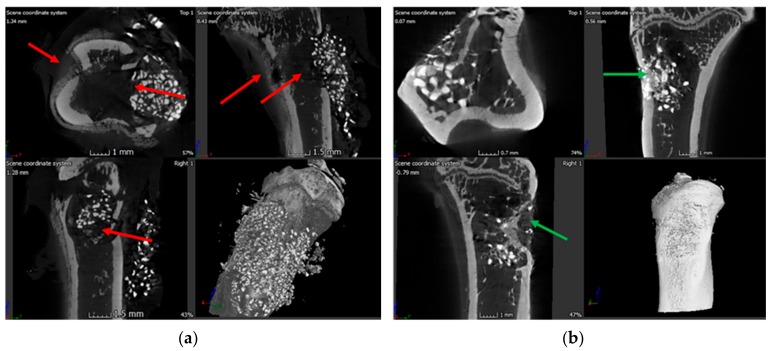

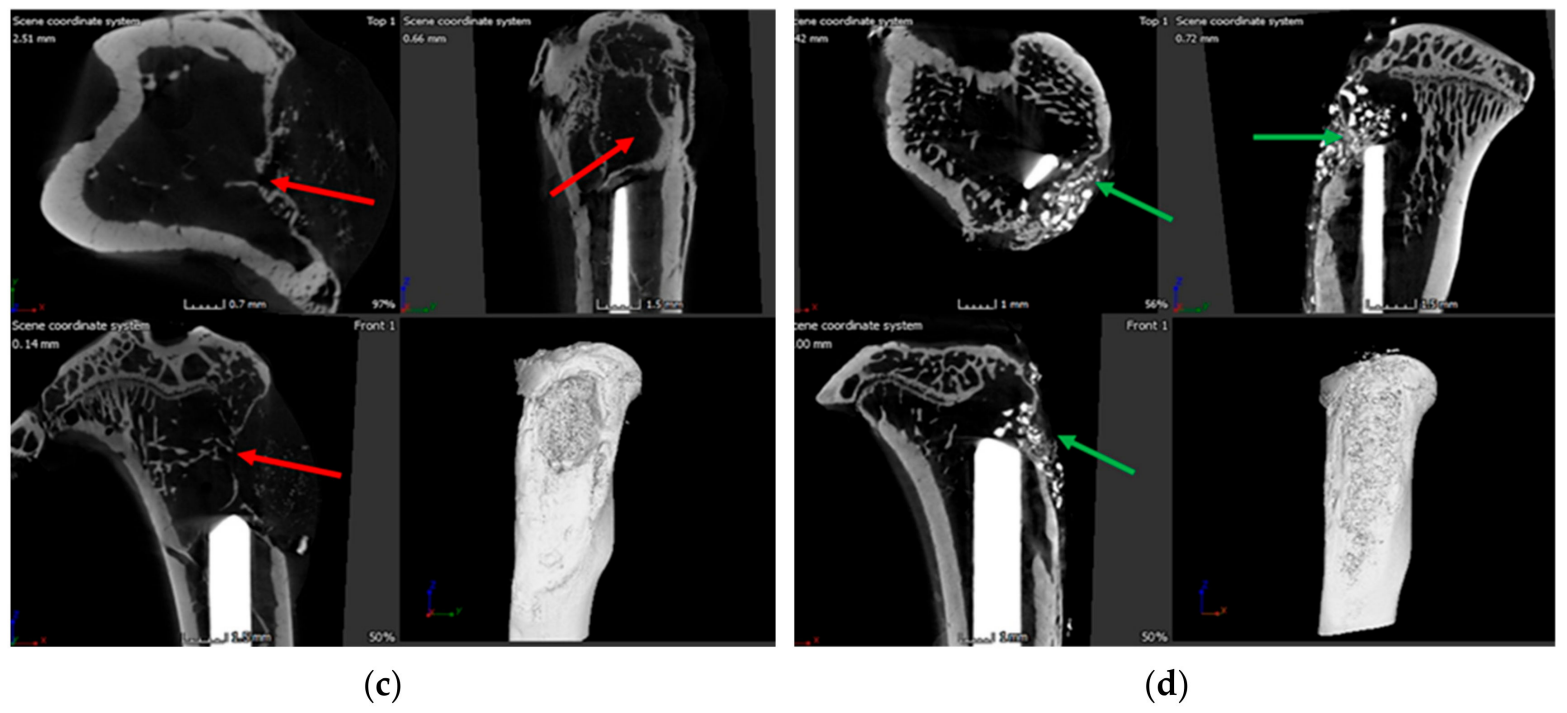
(**a**) The μ-CT images of drill-hole rat control group demonstrated signs of osteomyelitis through signs of infection, decreased bone formation and formation of sinus tract. Infection seemed to have spread to the posterior of the bone. (**b**) The drill-hole treatment group showed no signs of osteomyelitis and the bone appeared to be healed with new bone formation and cortical bone bridging. Bone seemed to be undergoing remodeling. (**c**) The μ-CT images of k-wire rat control group demonstrated signs of osteomyelitis through signs of infection, decreased bone formation and formation of sinus tract. (**d**) The k-wire treatment group showed no signs of osteomyelitis and the bone appeared to be healed with new bone formation. Bone seemed to be undergoing remodeling.

X-ray and μ-CT were conducted to assess the bone health. X-ray imaging of cohort 1 animals showed signs of osteomyelitis with formation of sequestrum, abscess, lytic regions and periosteal thickening (in Supplementary Figure S3). Similar radiologic features were seen in the k-wire control group, with clear signs of osteomyelitis through regions of periosteum in addition to lysis of bone, loss of cortical bone and presence of necrotic bone (Figure 5a). By contrast, x-ray images of rats in the k-wire treatment group in cohort 4, did not show any sign of infection (Figure 5b). Regardless of the cohort, the k-wire seemed well positioned in the intramedullary space. No adverse events were observed. μ-CT of cohort 1 (Figure 6a) showed widespread signs of infection. Specifically, these images showed the presence of sinus tracts and fibrous material (red arrows). Finally, infection looked to have spread with the posterior tibia showing signs of osteolysis and cortical destruction. Similarly, bones in cohort 3 rats (Figure 6c) also displayed sinus tract formation and a narrowing of the marrow space. Cortical bone growth with periosteal thickening was also evident at places. A large fibrous capsule was noticeably covering the bone defect. Conversely, cohort 2 rat bones (Figure 6b) showed healing of bone and cortical bridging. The bone appeared to be undergoing remodeling. Likewise, cohort 4 rat bones (Figure 6d) appeared to have new bone integrated with implanted ABVF (i.e., osseointegration). Alternatively, in this cohort, bone healing seemed more advanced.

### 3.7. Bone Volume of Newly Formed Bone

**Figure 7:**
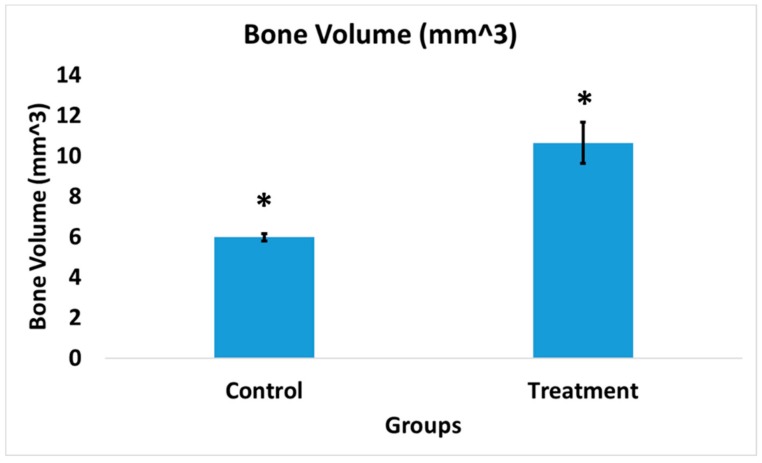
Bone volume in the infection control group of drill-hole model was significantly lower compared to the treatment group; *n* = 3 (control), *n* = 2 (treatment) * *p* < 0.02.

The bone volume of cohorts 1 and 2 was measured due to the higher sample size and longer duration of the study period that allowed a more accurate interpretation of the comparison. The treatment group showed significantly higher (*p* < 0.02) bone volume at the surgical site (ROI) compared to the control group (Figure 7). The bone volume of cohort 1 was 5.98 ± 0.18 mm^3^. In contrast, the cohort 2 treatment group exhibited a much higher bone volume of 10.65 ± 1.03 mm^3^.

### 3.8. Histology

**Figure 8:**
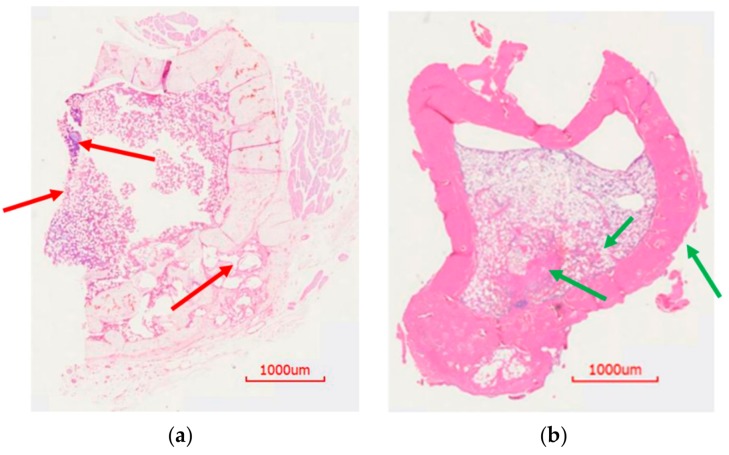
H&E staining of rat bones from drill-hole model. (**a**) H&E staining of control rat bone. There was a lack of bone growth at the dilled site. There was some destruction of the bone. (**b**) Bone from treatment group rat showed healing of the bone and cortical bridging with new bone growth.

**Figure 9:**
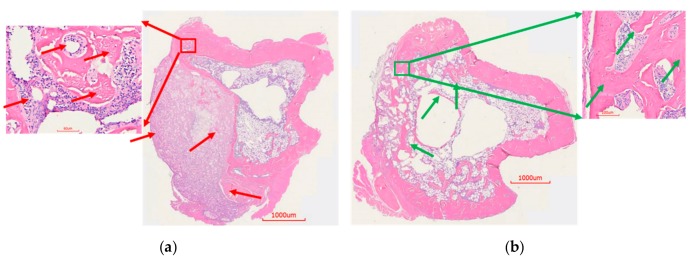
H&E staining of rat bones from k-wire osteomyelitis model. (**a**) H&E staining of control rat bone. There was bone growth but with narrowing of marrow space. In places, there was thickening of bone. A big fibrous capsule with inflammatory cells was visible. (**b**) Bone from the treatment group rat showed ongoing healing of the bone. New bone formation was visible in the defect. Osseointegration was seen as the degradation of the ABVF allowed new bone to grow in.

To assess the histological features of the bone at the surgical site, H&E staining was done. Cohort 1 showed unhealed bone, lack of new bone growth, and the presence of fibrous tissue as a consequence of infection (red arrows, Figure 8a). On the contrary, cohort 2 showed healed bone with cortical bridging, new bone growth and osseointegration, likely resulting from the absence of infection (Figure 8b). Accordingly, cohort 3 animals were consistent in showing signs of massive infection. Osteomyelitis was evidenced by large fibrous capsules with an abundance of inflammatory cells, narrowing of the marrow space, and a combination of thickening and destruction of boney structure (red arrows, Figure 9a). Conversely, histology of the cohort 4 showed new bone formation, ongoing bridging of newly formed bone and limited mature collagen structure (green arrows, Figure 9b). These signs are consistent with the documented healing process of bone and absence of infection.

### 3.9. Bacterial Colony Count

**Figure 10:**
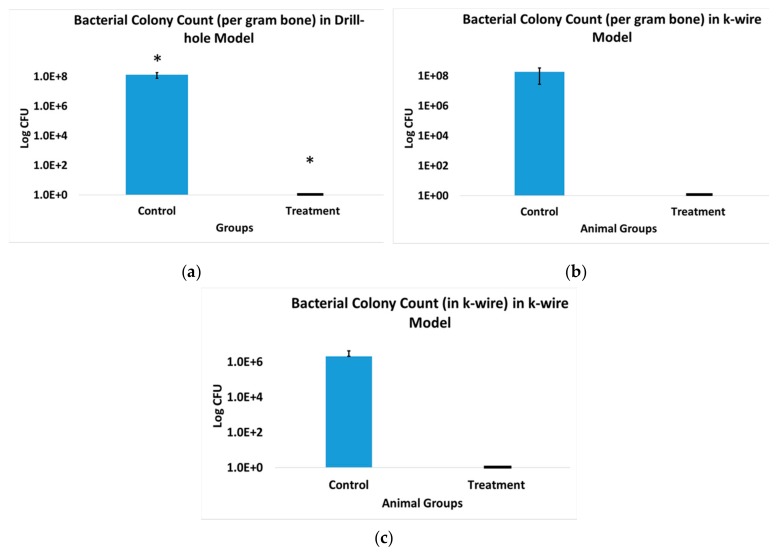
(**a**) The bacterial count in the drill-hole model control group showed very high bacterial count in the bone; *n* = 4 (control), *n* = 3 (treatment), * *p* < 0.05. The treatment group did not show any presence of bacteria and the infection was eliminated. (**b**) The bacterial count in the k-wire rat model control group showed very high bacterial count in the bone. The treatment group was free from any bacterial load and the infection was eliminated; *n* = 2 (control), *n* = 2 (treatment). (**c**) The bacterial count in k-wire rat model control group showed a high bacterial count on the extracted k-wire. The k-wire extracted from the treatment group was free from any bacterial load; *n* = 2 (control), *n* = 2 (treatment). *S. aureus* (ATCC 49230) was used for all the experiments.

The bacterial colony counts revealed a high bacterial content (1.33 × 10^8^ ± 5.56 × 10^7^ CFU/gm bone) in cohort 1 rats that did not receive ABVF (Figure 10a), whereas, complete obliteration of bacteria was seen in cohort 2 treatment rats; no bacterial colonies were observed after culturing (Figure 10a), showing a statistically significant reduction in bacterial content (*p* < 0.05). Similarly, high bacterial loads were also present in cohort 3 rats (Figure 10b), in contrast to a distinct lack of bacteria and infection in the treatment group (cohort 4, Figure 10b). Bacterial content on the k-wire extracted from cohort 3 showed a high mean bacterial load of 2.03 × 10^6^ CFU/k-wire (ranged between 1 × 10^5^ to 3.95 × 10^6^ CFU/k-wire) (Figure 10c). No bacteria were able to be grown from the k-wires extracted from cohort 4 rats (Figure 10c), indicating the absence of bacteria and infection.

## 4. Discussion

In spite of the many disadvantages of PMMA-based bone cement, such as non-biodegradability, poor drug release kinetics, immune response and additional surgical removal, etc., it is still the standard of care for treating infected TJR [27,28]. FDA-approved bone cements generally use tobramycin, gentamicin and vancomycin as the antibiotic payload [29], although none of these antibiotics are effective against bacterial biofilms [30]. The efficacy of aminoglycoside antibiotics may be hindered by the (1) low metabolic activity of bacteria in a biofilm, (2) poor ability to penetrate into the biofilm through extracellular polysaccharide matrix, and (3) insufficient and non-uniform distribution of antibiotics within the biofilm [31,32,33]. Furthermore, the presence of biofilm bacteria, which are not actively dividing and are in a low metabolic state, may also render vancomycin less effective since vancomycin targets the cell wall of bacteria by blocking cell wall synthesis [34]. The limited efficacy of vancomycin against biofilm was documented both in vitro and in vivo [30]. Local delivery of antibiotic is needed to effectively treat the TJR related osteomyelitis, as systemic antibiotic cannot reach high enough concentration in dense cortical bone as well as in avascular bone. No viable options to deliver antibiotic, apart from bone cements, are regularly used in patient treatment [29]. Moreover, antibiotics such as rifampicin, which is known to be active against biofilm bacteria, cannot be incorporated in bone cement as it hinders the polymerization of PMMA bone cement, compromising the material properties of PMMA [35]. 

The incompatibility of rifampicin with PMMA significantly limits the efficacy of PMMA in cases of implant-associated biofilm formation, a common occurrence in TJR. Previous studies have indicated that bacterial biofilm formation can happen within 5–10 h following bacterial seeding in wound [36]. Moreover, the presence of blood plasma can potentially enhance the propensity of *S. aureus* to form a biofilm. Unlike vancomycin, which has limited or no activity against *S. aureus* biofilms [30,37], active replication of bacteria is not needed for rifampicin activity, making it an effective alternative for biofilm bacteria [38]. Rifampicin is not only active against *S. aureus* biofilms but also active against *S. epidermis* bacterial biofilm, another common causative agent of implant related osteomyelitis [39]. Unfortunately, bacteria can readily develop resistance to rifampicin due to its mode of action. Thus, combination therapy with another antibiotic is necessary. Vancomycin, which is effective against planktonic *S. aureus* infection, can be delivered as a cocktail with rifampicin, to provide better therapeutic outcome. The synergistic effect of rifampicin in combination with vancomycin has been previously documented [40]. Other aminoglycoside antibiotics, like tobramycin showed no beneficial outcome with rifampicin, while others such as gentamicin seem to be antagonistic [40]. A rifampicin-vancomycin combination could be a viable weapon in our therapeutic arsenal against *S. aureus* biofilm infection. 

As expected from other reports in the literature, the drug release media showed efficient bioactivity with good zones of inhibition (Figure 6) over 6 weeks. Based on our previous work in which we validated that a mature *S. aureus* biofilm could be reliably formed within 24 h of bacterial seeding, is also supported by other studies [34,41]. *S. aureus* biofilms were exposed to drug release media, which showed complete eradication of viable bacteria within a biofilm both in a well-plate and on a k-wire. This result is supported by previous studies, where this antibiotic combination showed bacterial killing within a biofilm [42,43]. The in vivo experiments also showed complete eradication of viable bacteria from the biofilm in the bone and on the k-wire of cohort 4 (treatment group), as no bacteria could be cultured after extraction. In the cohort 3 control group, not only was the bacterial load very high in the bone, but it was also very high on the k-wire in all the rats. Although no statistically significant difference was found between cohorts 3 and 4 due to the preliminary nature of this part of the study, it can be noted that bacterial elimination was achieved in the cohort 4 treatment group in the rats. Additionally, it is worth noting that the standard deviation of colony counts on the k-wire was extremely high, likely the result of a partial delamination of the biofilm when extracting it from the intramedullary space. Nevertheless, in the absence of antibiotic treatment, all the k-wires had high bacterial load. Similarly, bones of the control cohorts showed high bacterial loads, although the variability of the load was high, ranging from 5.42 × 10^7^ to 3.35 × 10^8^ CFU/gram bone. Not only could delamination have impacted this range, but more importantly, the presence of an encapsulated, purulent-filled cavity in the bone of the control animals made “sterile” processing difficult, resulting in a puncture of the capsule and the loss of some CFUs. Nevertheless, all the control animals showed infection, whereas treated animals were free from infection. Finally, despite the clear difference in treatment and control animals in this preliminary study, statistical significance was not achieved, likely due to the factors outlined above. However, the study was extremely promising and based on the effect size in these cohorts (i.e., incidence of infection in cohort 3 = 100% and incidence of infection in cohort 4 = 0%) and a post hoc continuous power analysis of the means and standard deviations found in the colony counts, a sample size of 11 rats in each of cohort 3 and 4 would provide a statistically significant result (α = 0.05 and power of 0.8). 

Although the HPLC only detected the presence of drugs until week 2 in a sustained manner, it is clear that the drug release was sustained until week 6 based on the bioactivity of the released drug in the ZOI study. The limited detection of the HPLC method was likely a reflection of interference from polymer degradation products, and further method development may be needed to detect the drugs. Nevertheless, HPLC detected the cumulative release of 30.41% of the total incorporated vancomycin and 14.38% of the incorporated rifampicin over the course of two weeks (Figure 1). 

Although vancomycin has limited reported cytotoxicity and is well tolerated by osteoblasts even at high concentration, rifampicin has reported toxicity to osteoblast cells [44]. A decrease of up to 50% viability can be seen at 5 mg/mL of rifampicin, whereas vancomycin has no reported cytotoxicity at that concentration [44]. This may explain the reduced viability of osteoblasts when exposed to released drug from ABVF (Figure 4) and raises concerns that rifampicin may hinder in vivo bone regeneration. However, such an effect was not observed in vivo in our study. On the contrary, our ABVF putty was able to support bone healing and osseointegration (Figure 6, Figure 8 and Figure 9). Furthermore, this finding shows congruence with a recent study where the application of rifampicin powder in a sterile wound did not result in reduced healing of bone. In addition, rifampicin in an infected wound bed showed faster healing of bone compared to the no antibiotic control and the vancomycin-only treated group previously reported [37]. Furthermore, in the current study, the rifampicin/vancomycin cocktail showed an absence of signs of infection in the biofilm infection model (Figure 6 and Figure 9), whereas the control group showed osteolysis, lucency, and the presence of bacteria (Figure 6, Figure 9 and Figure 10). These findings highlight the lack of exact translation between in vitro and in vivo outcomes, perhaps because natural cellular turnover and renewal provides some additional resistance to the cytotoxic effects observed in vivo. Importantly, the presence of bacteria can reduce the number of osteoblasts and increase osteoclastic resorption by inciting inflammation. Additionally, native pluripotent stem cells may lose the ability to differentiate into osteoblasts if exposed to bacteria [45]. Thus, it is wise to take measures to eliminate bacteria first as a treatment priority to allow the body’s innate bone healing mechanisms in the absence of infection and inflammation. 

Although vancomycin has limited to no known cytotoxicity, it can be a source of nephrotoxicity during prolonged, systemic high doses. To validate that the local delivery of vancomycin from the ABVF putty did not also invoke such a response, we carried out the serum creatinine assay (data not shown) and did not see any signs of increased creatinine in the rat’s serum, proving the absence of nephrotic adverse response due to vancomycin released from ABVF. In literature, another antibiotic used in PMMA bone cement, tobramycin, showed significant nephrotoxicity at 80 mg/kg/day [46]. This was not surprising based on the fact that 200 mg/kg/day of vancomycin has been demonstrated to be safe without nephrotoxic effects [46] and the total vancomycin incorporated in our ABVF is well below the nephrotoxic dose. Hence, local delivery of vancomycin not only prevents high systemic exposure, but incorporation into ABVF resulted in a much lower amount of vancomycin being used. 

Alternatively, toxicity related to rifampicin dosing, including gastrointestinal, hepatic, renal, hematological, and central nervous system effects, has been reported, with doses ranging from 9 to 15 g of drug. Furthermore, systemic dosed rifampicin has been reported to be 70–80% protein bound and widely distributed throughout the body [23]. Nevertheless, rifampicin is a critical antibiotic for treating prostheses biofilm infections, especially when the hardware is retained [47]. Previous attempts to use rifampicin alone or in combination with other antibiotic such as vancomycin showed better antibacterial activity but failed to totally eradicate the bacterial load both in bone and in biofilm [48,49]. This may have been caused by insufficient drug release kinetics and in some cases, the inability to incorporate sufficient amounts of antibiotic in the delivery device [35,49]. In our study, ABVF showed complete eradication of both a planktonic bacterial load as well as bacteria in biofilm (Figure 3 and Figure 10) both in vitro and in vivo.

## 5. Conclusions

The successful infection eradication in rats and obliterated biofilm on the k-wire shows the promise of a dual antibiotic releasing ABVF to treat TJR related infections, enabling surgeons to retain the prosthetic implant. Furthermore, the developed dual release ABVF putty may also enable surgeons to perform single-stage revision surgeries, reducing the immense burden on the healthcare system.

ABVF provided effective release of rifampicin and vancomycin, showing in vitro and in vivo efficacy by eradicating the bacteria. ABVF was biodegradable and provided support for new bone growth. No adverse effect on in vivo bone healing was observed. No apparent toxicity was also seen in the rats.

## Figures and Tables

**Table 1:** Description of the cohorts used in this study.

Cohort	Implant	Surgery Model	Infectious Dose	Time to Sacrifice (Weeks)	Cohort Purpose	Morbidity/Infection	Cohort Size
1	BVF putty without antibiotics	Drill-hole Osteomyelitis	10^8^ CFU	10	Positive Infection control	Local infection	12
2	ABVF with vancomycin and rifampicin	Drill-hole Osteomyelitis	10^8^ CFU	10	Infection treatment	No infection	5
3	BVF putty without antibiotics	K-wire biofilm osteomyelitis model	Biofilm on k-wire	6	Positive Infection control	Local infection	3
4	ABVF with vancomycin and rifampicin	K-wire biofilm osteomyelitis model	Biofilm on k-wire	6	Infection treatment	No infection	3

## Supplementary Materials

The following are available online at https://www.mdpi.com/1999-4923/11/11/592/s1, Figure S1: The zone of inhibition study done with released drug in 2 mL and 5 mL release media showed similar antibacterial activity against *S. aureus* (ATCC 49230), Figure S2: The zone of inhibition study was done with (a) vancomycin and (b) rifampicin standards to see activity against *S. aureus* (ATCC 49230). For vancomycin, concentration of ≥0.5 µg/mL showed ZOI indicating antibacterial activity. For rifampicin, concentration of ≥0.031 µg/mL showed ZOI indicating antibacterial activity, Figure S3: The x-ray images of cohort 1 rat tibia (control group rat in drill-hole model). Bone with osteomyelitis is visible.
