# Supplementary material for: Extended Release Combination Antibiotic Therapy from a Bone Void Filling Putty for Treatment of Osteomyelitis
Source: Pharmaceutics. 2019 Nov 8;11(11):592. doi: 10.3390/pharmaceutics11110592 (PMC6920883; doi:10.3390/pharmaceutics11110592)
Supplement: Supplementary file 1 [file pharmaceutics-11-00592-s001.pdf]

pharmaceutics

Supplementary Materials: Extended Release Combination Antibiotic Therapy from a Bone Void Filling Putty for Treatment of Osteomyelitis

Raquib Hasan, Kambri Schaner, Meredith Schroeder, Abbey Wohlers, Jacob Shreffler, Codi Schaper, Hariharaputhiran Subramanian and Amanda Brooks

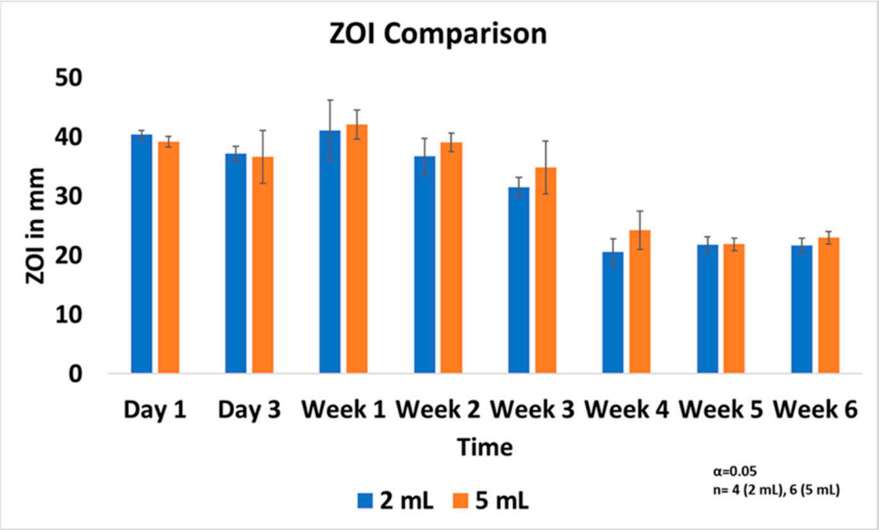

Figure S1. The zone of inhibition study done with released drug in 2 mL and 5 mL release media showed similar antibacterial activity against *S. aureus* (ATCC 49230). This may indicate that changes in release media volume did not alter the drug release from ABVF and that sink condition was maintained.

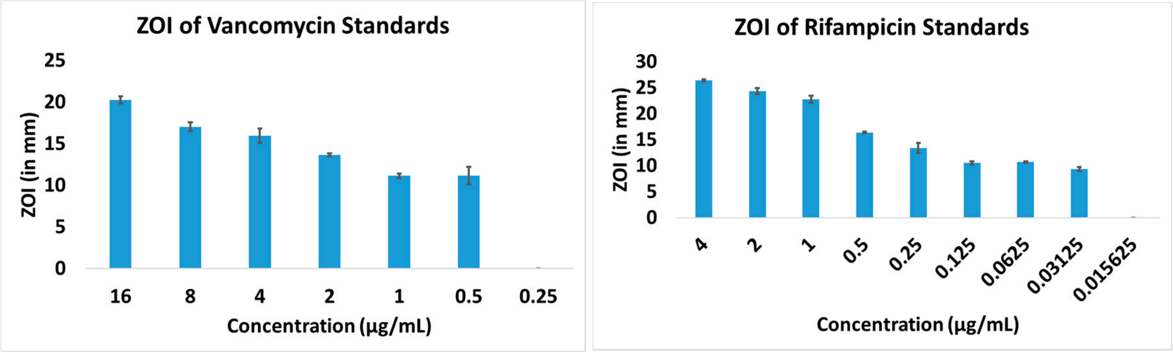

(a) (b)

Figure S2. The zone of inhibition study was done with (**a**) vancomycin and (**b**) rifampicin standards to see activity against *S. aureus* (ATCC 49230). For vancomycin, concentration of ≥0.5 µg/mL showed ZOI indicating antibacterial activity. For rifampicin, concentration of ≥0.031 µg/mL showed ZOI indicating antibacterial activity.

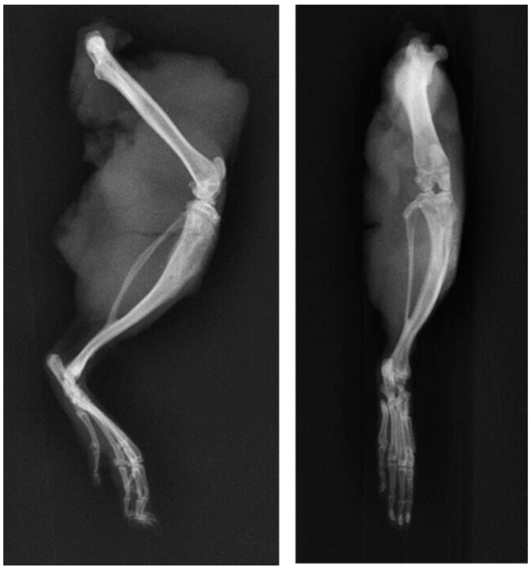

Figure 3. The X-ray images of cohort 1 rat tibia (control group rat in drill-hole model). Bone with osteomyelitis is visible.
